# Efficacy Analysis of the Reinforcing and Circulation-Promoting Protocol of Acupuncture and Moxibustion in Treatment of Twenty-Four Patients with Refractory Chronic Low Back Pain

**DOI:** 10.1155/2022/8734207

**Published:** 2022-04-23

**Authors:** Jincao Zhou, Jingjing Wang, Zhongjie Chen, Mozheng Wu, Yue Jiao, liyun He

**Affiliations:** ^1^Institute of Acupuncture and Moxibustion, China Academy of Chinese Medical Sciences, Beijing 100700, China; ^2^Hospital of Tsinghua University, Beijing 100084, China; ^3^Institute of Basic Research in Clinical Medicine, China Academy of Chinese Medical Sciences, Beijing 100084, China

## Abstract

**Objectives:**

The purpose of this study was to observe the clinical efficacy of the reinforcing and circulation-promoting protocol of acupuncture and moxibustion in treatment of refractory chronic low back pain, analyze therapeutic principles to obtain treatment efficacy, and develop new therapeutic principles to treat chronic low back pain.

**Methods:**

Twenty-four patients from the registry of patients suffering from refractory chronic low back pain were invited to our self-controlled case series, which was conducted in “real-world” settings. We implemented the reinforcing and circulation-promoting protocol of acupuncture and moxibustion to treat these patients and used the Visual Analogue Scale (VAS) and the Oswestry Disability Index (ODI) as the observation indices.

**Results:**

All 24 patients completed the treatment of acupuncture and moxibustion. The VAS of low back pain was 6.83 ± 2.18 before treatment and 2.13 ± 1.45 after treatment. The difference before and after treatment was significant (*P* < 0.001). The ODI was 28.21 ± 13.06 before treatment and 16.63 ± 7.20 after treatment. Their difference before and after treatment was significant (*P* < 0.001).

**Conclusion:**

The reinforcing and circulation-promoting protocol of acupuncture and moxibustion is effective in treating refractory chronic low back pain mainly because low back pain can be significantly relieved and motor function can be promoted. This trial was registered with AMCTR-OOO-17000045 (3 December 2016).

## 1. Introduction

Chronic low back pain (CLBP) is one of the most common chronic pain problems in the world. In Medical Subject Headings ((MeSH), CLBP is defined as “acute or chronic pain in the lumbar or sacral regions, which may be associated with musculo-ligamentous sprains and strains, intervertebral disc displacement, and other conditions” [1]. Among common chronic lumbar diseases are lumbar muscle strain, prolapse or protrusion of the lumbar intervertebral disc, hypertrophic spondylitis, ankylosing spondylitis, and old lumbar fracture. An estimated 84% of people experience chronic low back pain during their lifetime, and many of them will experience a recurrent episode [2]. Globally, the rate of disability related to CLBP is much higher than that related to other diseases. This leads to pressure on the society and medical care, which cannot be ignored. [3] Unfortunately, the consensus among many countries is that the efficacy of CLBP treatment is good in the short term but diminishes over longer periods [4, 5]. There are pharmacological and nonpharmacological interventions for treating CLBP. Nonpharmacological interventions account for 50%, including sports activities, patient education, psychological intervention, manual treatment, alternative therapies, and medical equipment, while pharmacological interventions include analgesics, muscle relaxants, steroids, antidepressants, antiepileptics, vitamins, and supplements [2]. To date, these treatment methods of this disease have not produced satisfactory results. Normally, acute low back pain improves markedly in the first six weeks after treatment, but two-thirds of patients will still have pain in the following three months [6, 7]. Furthermore, a recurrence occurs to one-third of the patients within one year [8]. In the past twenty years, although the U.S. and European countries have published many guidelines for clinical diagnosis and treatment of CLBP, only 20% of them recommend acupuncture-moxibustion as a therapeutic method [9]. These guidelines provide neither much information about acupuncture and its types nor many reports on observing the clinical efficacy over long periods. In addition, the guidelines present many observational studies that were conducted based on a type of syndrome differentiation because they were restricted by strict experimental conditions. Many observational studies also merely focused on the treatment phase, and consequently, they lack integrity.

In China, the practice of acupuncture-moxibustion to treat CLBP has a long history. First recorded in Prescriptions for Fifty-two Diseases (Wushier Bingfang) is the assertion that a problem with the bladder meridian of foot-taiyang can cause the back, low back, and gluteal region to ache [10]. Chapter 41 of Plain Questions (Suwen) specifies the treatment of low back pain. In addition, Chapter 10 of Miraculous Pivot (Lingshu) and chapters 60 and 63 in Plain Questions propose using the nine classical needles to treat different types of low back pain due to varying causes. Having researched fifty-two ancient medical books, Zhu Shaobing et al. [11] concluded that deficiency syndrome, including kidney, liver, and spleen deficiencies, and/or excess syndrome due to, for example, wind, cold, dampness, heat, and stagnation, can lead to obstruction of meridians and collaterals, thereby causing low back pain. Their conclusion accords with the CLBP pathogenesis of deficiency in origin and excess in superficiality.

It is documented in many ancient medical books that CLBP was treated by puncturing a single distant point. Much of the medical literature in the late twentieth century describes the practice of distant–local point combination. Mainly used were points in the bladder meridian of foot taiyang, the governor vessel, and the gallbladder meridian of foot shaoyang, five transport points, crossing points, and back-shu points. However, at present, CLBP is often treated with acupuncture on Ashi and Jiaji points. Since the treatment principle of treating CLBP with acupuncture on Ashi points and points along the affected meridian is widely followed, currently, many traditional Chinese medicine (TCM) practitioners treat the pain by puncturing points in the back, low back, and lower limbs while ignoring the points on the abdomen and nonaffected meridians.

In terms of acupuncture therapy, TCM practitioners normally use traditional needling techniques such as the filiform needle therapy, bloodletting, and fire needling and modern techniques such as electroacupuncture and myofascial trigger points [12, 13]. In addition, they mainly use the reducing manipulation to treat tender points and observe short-term efficacy [14, 15]. However, such treatment strategies do not accord with the CLBP pathogenesis of deficiency and excess in complexity. This can point to the high recurrence rate of CLBP and unsatisfied treatment efficacy in the long term [4, 5]. It also suggests that we may be able to understand the pathogenesis of CLBP from the TCM perspective and adjust the current treatment strategies to propose more effective therapies. Existing studies have shown that in terms of improvement in pain relief, other subjective feelings, symptoms, and daily living skills, the effectiveness of using points on the abdomen and back, namely, the yin aspect and yang aspect, is better than that of the routine point combination [16–18].

According to the Agency for Healthcare Research and Quality [19], “a patient registry is an organized system that uses observational study methods to collect uniform data (clinical and other) to evaluate specified outcomes for a population defined by a particular disease, condition, or exposure and that serves one or more predetermined scientific, clinical, or policy purposes.” In other words, a patient registry is a collection, for one or more purposes, of standardized information about a group of patients who share a condition or experience. To observe the clinical efficacy of the reinforcing and circulation-promoting protocol of acupuncture and moxibustion in treatment of refractory chronic low back pain, we invited twenty-four patients from a chronic low back pain registry to our self-controlled case series (SCCS). These patients shared common symptoms such as severe pain and were expected to need long periods of complex treatment. Generally, patients with refractory CLBP can have different clinical manifestations, but from the TCM perspective, its pathogenesis can be reduced to insufficiency of healthy qi complicated by excessiveness of pathogenic qi. According to the TCM therapeutic principle of same treatment for different diseases, we treated these patients with similar techniques of acupuncture-moxibustion since they had the same pathogenesis, although they had different clinical manifestations. The deficiency syndrome of CLBP is mainly characterized by kidney, liver, and spleen deficiencies, and at the same time, one or multiple excess pathogens, including wind, cold, dampness, heat, and stagnation, contribute to CLBP. Instead of using the randomized controlled trial (RCT) in which a treatment is applied, we conducted a real-world study (RWS) that allowed us to apply different therapies to treat our patients since their pathogenesis was complicated. In our real-world study, based on the CLBP pathogenesis of deficiency and excess in complexity, we punctured points on the abdomen and back, followed the principle of simultaneous elimination and reinforcement during acupuncture, and applied the reinforcing and reducing manipulations. Besides, due to the complicated CLBP pathogenesis, we selected a suitable acupuncture therapy and puncturing order for each of the subjects. In this paper, we present our observation of the clinical efficacy of treating patients suffering from refractory CLBP with the reinforcing and circulation-promoting protocol of acupuncture and moxibustion and analyzed therapeutic principles to obtain treatment efficacy.

## 2. Materials and Methods

### 2.1. Clinical Data

This study was reviewed by the Ethics Committee of the Institute of Basic Research in Clinical Medicine, China Academy of Chinese Medical Sciences (CACMS), through 2016NO.13. It was registered on 3 December 2016 at the Chinese Clinical Trial Registry (ChiCTR–OOC–17010751) and the Acupuncture and Moxibustion ClinicalQ3 32 Trial Registry (AMCTR–OOO–17000045).

#### 2.1.1. General Information

The data were collected between 2017 and 2021. Twenty patients were hospitalized because they had obvious pain and motor impairment, while four were treated in the outpatient department. These four patients had pain and motor impairment as much as the inpatients. It was more difficult to treat them since they had high workload and sat or stood for a long time and consequently did not have good rest during the period of treatment (see Table 1).

#### 2.1.2. Diagnostic Criteria

According to Evidence-based Guidelines of Clinical Practice with Acupuncture and Moxibustion (2014) published by the China Association of Acupuncture and Moxibustion, Diagnosis and Treatment of Low Back Pain (2007) by the American Medical Association and American Pain Society, and Low Back Pain and Sciatica in Over 16s: Assessment and Management (2016) by the National Institute for Health and Care Excellence, the diagnosis of CLBP is based on the following criteria:

1. 12 weeks of pain or muscle tension below the chest and above the gluteal region, no matter whether there is pain in the lower limbs
2. Diagnostic information including determined causes, medical history, symptoms, physical examination, and/or imaging data.

#### 2.1.3. Inclusion Criteria

Those who were eligible for clinical observation should meet the following conditions: (1) they had a chief complaint of CLBP and met the diagnostic criteria mentioned above, (2) they were between 16 and 65 years old, (3) they had an acute stage and/or subacute stages of CLBP, (4) they were willing to receive acupuncture treatment, and (5) they were willing to participate in the case registration study and signed the informed consent.

#### 2.1.4. Exclusion Criteria

Patients were excluded if they had one of the following conditions: (1) they had a severe systemic disease such as a primary disease of the cardiovascular system, liver, kidney, or hematopoietic system, (2) they had severe psychosis, and (3) their low back pain was caused by tumor, infection, or trauma.

#### 2.1.5. Dropout of Patients

Patients were excluded if they could not complete the treatment due to personal reasons or received other treatment during our study period.

#### 2.1.6. Primary Conditions of Patients

We recruited twenty-four patients with low back pain and motor impairment as chief complaints, including seventeen cases with lumbar intervertebral disc degeneration and four cases who have a problem with the spine. Basic information of the patients' medical conditions and the types of pain they have are presented in Tables 2 and 3, respectively.

### 2.2. Criteria of Syndrome Differentiation

In this study, we adopted the criteria of syndrome differentiation described by the Chinese National Standard for Diagnosis and Curative Effect of Syndromes in Traditional Chinese Medicine and Diagnostics of Traditional Chinese Medicine. Our practitioners who had had relevant training used the criteria to conduct syndrome differentiation, namely, to identify deficiency syndrome and excess syndrome. The excess syndrome includes obstruction of cold-dampness, qi stagnation and blood stasis, obstruction of dampness-heat and cold-dampness, and blood stasis. The deficiency syndrome is assessed based on the following clinical manifestations: (1) the duration of the disease was long, (2) acute pain transitions to chronic pain, (3) the pain accompanied by soreness, numbness, and heaviness in the lumbus is referred to the lower limbs, (4) the pain may be dull, (5) the pain can be relieved by warmth and pressure, (6) the muscles of the lumbar and lower limbs are soft and weak, (7) the pain can be aggravated by tiredness and cold invasion and sometimes accompanied by soreness and weakness in the lumbus and knees, (8) a patient suffers from mental fatigue, (9) a patient has a slightly or dark red tongue as well as scanty coating, and (10) a patient's pulse is either deep, thready and weak, or thin and string-taut.

The excess syndrome is assessed based on the following clinical manifestations:

1. Obstruction of cold-dampness: most of the patients with this syndrome have experience of exposure to wind, cold, and damp conditions. They have pain and sensation of heaviness or cold in their lumbus. They may also have difficulty bending forward or backward. In addition, the pain is paroxysmal and can be referred to the lower limbs and aggravated by cold, wind, or rainy weather. The patients have a white and greasy tongue coating. Their pulses may be superficial and tense, deep, or deep and tense.
2. Qi stagnation and blood stasis: most of the patients with this syndrome have experience of weight bearing, meridian damage, sudden strain of a muscle, qi stagnation, blood stasis, and acute lumbar sprain and have experience working for a long time bending forward. The patients have back stiffness and stabbing pain in the fixed location of their lumbus. The pain is aggravated by pressure. They have a dark red tongue or a tongue with ecchymosis. In addition, their pulse is chopping or string-taut and chopping.
3. Cold-dampness and blood stasis: palpation can detect subcutaneous nodules, cysts, steaks, and cold spots and swollen, cold, and stiff soft-tissues in painful areas.
4. Obstruction of dampness-heat: patients with this syndrome have pain with burning sensation in their lumbus and weakness in their lower limbs. The pain is aggravated by warmth or rainy weather but relieved by exercise. A patient's thirst is induced by heat. Their urine is scanty and deep yellow. Their tongue coating is yellow and greasy. Their pulse is either soft and rapid or string-taut and rapid.

### 2.3. Basic Principles of Treatment and Acupoint Selection

During treatment, we combined the reinforcing method with the circulation-promoting method. However, based on syndrome differentiation of deficiency and excess, we primarily used either of them. The main points we selected were back-shu points and points in the governor vessel and related meridians.

We used the reinforcing method to treat patients with the deficiency syndrome. We mainly punctured points on the abdomen by the filiform needle therapy to tonify the consumption and regulate the circulation of blood and qi between zang-fu organs. On the other hand, we used the circulation-promoting method to treat patients with the excess syndrome. We mainly selected Ashi points. After we had identified the nature of a pathogen, we used one or several techniques of acupuncture-moxibustion. Specifically, we used warming needle moxibustion or moxibustion to treat our patients with obstruction of cold-dampness. For patients with qi stagnation and blood stasis, we applied the filiform needle therapy to regulate the circulation of qi and activate blood. For patients with cold-dampness and blood stasis, we applied the fire needling therapy or warming needle moxibustion not only to expel cold-dampness and blood stasis but also to warm and unblock meridians and collaterals. In addition, we used the bloodletting cupping therapy to treat patients with damp-heat obstruction.

#### 2.3.1. Acupoint Selection

Basically, we selected points from the back-shu points, points in the governor vessel, and points along the affected meridians. To be specific, the back-shu points include Pishu (BL20), Shenshu (BL23), Dachanghu (BL25), Sanjiaoshu (BL22), Qihaishu (BL24), Xiaochangshu (BL27), and Pangguangshu (BL28). According to the location of pain, we followed the treatment principles of covering the affected area and using as few acupoints as possible to select the back-shu points in the affected segment. The points normally selected were Pishu (BL20), Shenshu (BL23), Dachangshu (BL25), and Zhibian (BL54); points along the governor vessel include Jizhong (GV6), Mingmen (GV4), Yaoyangguan (GV3), and Shiqizhui (EX-B7). After having identified the syndrome of a patient, we followed the treatment principles of covering the affected area and using as few acupoints along the meridians as possible to select points along the governor vessel in the affected segment; points along the affected meridians refer to points along the meridians in the region of the lower limbs, in which pain and discomfort were localized. We often used points along the bladder, gallbladder, and stomach meridians, including Huantiao (GB30), Yanglingquan (GB34), Weizhong (BL40), Feiyang (BL58), Xuanzhong (GB39), Zusanli (ST36), and Sanyinjiao (SP6). After having identified the syndrome of a patient, we followed the treatment principles of covering the affected area and using as few points along the meridians as possible to select points along the meridians in the affected segment.

In addition, we used two acupoint selection methods to reinforce qi and blood and promote their circulation. To be specific, for reinforcement, we often selected Zhongwan (CV12), Xiawan (CV10), Qihai (CV6), Guanyuan (CV4), Zhongji (CV3), Daheng (SP15), and Tianshu (ST25). If the low back pain was referred to the hip joints, we added Wailing (ST26) to the affected side. If the pain was referred to the knees and legs, we added Zusanli (ST36) and Dubi (ST35) to the affected side. If the kidney yang of a patient was deficient, we added Dahe (KI 12), and if a patient had pain along the sides of their spine, we added points along the kidney meridian on the abdomen.

For promoting circulation, we often punctured Ashi points in the painful area. We applied the fire needling therapy to disperse subcutaneous nodules and cysts in the lumbus. If there was obstruction in meridians and collaterals, we applied the bloodletting cupping therapy. We applied warming needle moxibustion or fire needling to treat regional cold pain. In terms of regional cold pain and dampness, we used warming needle moxibustion or moxibustion. To remove qi stagnation and blood stasis, we often selected the shu points of five-shu points along hand yang meridians on the distal side of the hand and sometimes asked our patients to move during acupuncture.

#### 2.3.2. Operation

If a patient's pain was mainly caused by the excess syndrome, we used, primarily, acupuncture on points on their back and then, to a lesser extent, on points on their abdomen.

On the other hand, if a patient's pain was mainly caused by the deficiency syndrome, we used, primarily, acupuncture on points on their abdomen and then, to a lesser extent, on points on their back.

If a patient had a local pain point, cold spot, cyst, steak, or blue vein on their back, we appropriately applied bloodletting and cupping therapy, fire needling therapy, warming needle moxibustion, or moxibustion and then, to a lesser extent, used acupuncture with filiform needles on points on their abdomen or back to regulate the circulation of their qi.

During consolidation treatment, we used, primarily, acupuncture on points on the abdomen and then, to a lesser extent, on points on their back.

#### 2.3.3. Frequency of Treatment

Our patients had treatment 2 or 3 times a week until the pain was remarkably relieved, followed by 3 to 6 times of consolidation treatment.

#### 2.3.4. Index of Observation

We collected our data before and after the treatment using the Visual Analogue Scale (VAS) and the Oswestry Disability Index (ODI).

#### 2.3.5. Statistical Analysis

We carried out a two-tailed hypothesis test to analyze our data. We set our alpha to 0.05, so if the *p*-value was 0.05 or lower, the observed differences were considered statistically significant.

When we performed the descriptive statistics, we used measures including the mean, standard deviation, median, minimum and maximum variables, and interquartile range (Q3-Q1). In addition, our enumeration data were composed of categorical variables such as the age ratio and the percentage of patients with distending pain. When we compared the data collected before and after treatment, we used the paired *t*-test (normal distribution) and the Wilcoxon signed rank-sum test (non-normal distribution).

## 3. Results

In our real-world study, our twenty-four patients completed their treatment as required. We collected the data before and after the treatment using the VAS and the ODI. Then, we performed descriptive statistics, and our results had statistical significance. The descriptive statistics of the treatment period, treatment time, onset time, and improvement time are shown in Table 4. The treatment period refers to the total length of time over which our twenty-four patients underwent treatment. The onset time refers to the time when a patient's pain stopped aggravating or began alleviating, and the improvement time refers to the time when a patient's pain relief was apparent.

We compared the VAS pain scores collected before and after treatment and found that the results had statistical significance (*P* < 0.0001). The VAS pain scores after treatment were significantly lower than those before treatment (see Table 5). In addition, we compared the ODI scores collected before and after treatment and found that the results had statistical significance (*P* < 0.0001). The ODI scores after treatment were significantly lower than those before treatment (see Table 5).

## 4. Discussion

Based on the findings derived from the data analysis undertaken above, we will discuss how we should select acupoints, which order we should take for acupuncture, and how we should combine acupuncture therapies.

### 4.1. Emphasis on Selecting Points on the Abdomen to Accord with Pathogenesis

Guided by the theory of obstruction leading to pain, many TCM practitioners normally puncture points in the affected and adjacent areas to unblock the collaterals and relieve pain. Such treatment can provide pain relief in the local area in a short time. However, during treatment, if qi and blood are not reinforced and the functions of zang-fu organs are not regulated to tonify the consumption of zang-fu organs, treat muscle and tendon strain, and counteract the degeneration of muscles and tendons, the pain will recur soon. Therefore, the consumption of healthy qi should be considered as one of the main causes of CLBP recurrence. This accords with the TCM theory of poor nourishment leading to pain.

However, if we select, based on the meridian syndrome differentiation, points in the governor vessel, urinary bladder meridian of foot taiyang, and gallbladder meridian of foot shaoyang but do not achieve expected effectiveness, we can select tonic points in the conception vessel, spleen meridian of foot taiyin, stomach meridian of foot yangming, liver meridian of foot jueyin, and kidney meridian of foot shaoyin. Although these points are not in the affected area or the affected meridian, such treatment accords with the therapeutic principle of treating deficiency with tonification. In other words, by puncturing these points, yang can be conducted from yin and the healthy reinforced one to eliminate pathogens, thereby achieving great effectiveness. For thin patients with qi and blood deficiency, we should prescribe traditional Chinese medicine and then use, primarily, moxibustion but also, to a lesser extent, acupuncture. Besides, we should select as few acupoints as possible.

The yang ming meridian is rich in qi and blood. The spleen-stomach acts as the source of qi and blood. Therefore, puncturing tonic points, including Tianshu (ST25), Huaroumen (ST24), Wailing (ST26), Shuidao (ST28), Guilai (ST29), and Zusanli (ST36) in the stomach meridian of foot-yangming and Daheng (SP15) and Sanyinjiao (SP6) in the spleen meridian of foot-taiyin, can tonify Qi and blood, invigorate the spleen and stomach, and improve the function of the low back and knees. Strengthening the middle and lower jiao can promote blood circulation on the low back and abdomen. Qi circulates between the conception vessel and the governor vessel. Acupuncture on Zhongwan (CV12), Xiawan (CV13), Yinjiao (CV7), Qihai (CV6), and Guanyuan (CV4) can tonify the liver and kidney and enhance Qi. Therefore, the principle of conducting yang from yin can be followed to effectively treat the pain on the governor vessel at the center of the spine. The urinary bladder meridian is a main meridian in the low back. Zhongji (CV3) is the alarm point of the urinary bladder. The lumbus is the house of the kidney, so puncturing points on the urinary bladder meridian such as Huangshu (KI16), Qixue (KI13), Dahe (KI12), and Henggu (KI11) can tonify the kidney qi. Therefore, puncturing these points appropriately can help treat CLBP.

When applying abdominal acupuncture to treat CLBP, we should initially puncture the points for conducting qi back to its origin, namely, Zhongwan (CV12), Xiawan (CV13), Qihai (CV6), and Guanyuan (CV4), and appropriately add one or more acupoints including Daheng (SP15), Wailing (ST26), Guilai (ST29), Shuidao (ST29), and Zhongji (CV3). This treatment strategy corresponds to the traditional principle of using acupuncture for tonifying the kidney, spleen, and governor vessel and that of conducting yang from yin. Besides, this treatment can effectively handle the problem with the consumption and can thereby achieve great overall treatment effectiveness.

### 4.2. Order of Acupuncture according to Pathogenesis

During treatment, we can alternate puncturing points on the abdomen with puncturing points on the back. In addition, we should determine the order of acupuncture on the abdomen and back based on the state of increase and decrease between excess and deficiency.

According to Zhongshi (Lingshu), the prerequisite for treating a disease is to find its causes. Since CLBP recurrence or aggravation normally has clear inducing factors, we should remove the factors before carrying out routine treatment. For example, if external wind and cold lead to CLBP recurrence or aggravation, we should initially puncture Hegu (LI4), Fengchi (GB20), and Weizhong (UB40). If the inducing factor is sprain, we should appropriately puncture Ganshu (UB18), Geshu (UB17), Hegu (LI4), and Taichong (LR3) in the first place. To eliminate emotional factors, first, we should appropriately puncture Baihui (GV20), Shenmen (HT7), Neiguan (PC6), and Taichong (LR3). After removing the inducing factors, we can apply the three-edged needle or fire needle therapy to carry out routine treatment for patients who have good constitutions and symptoms in their low back, for example, fixed tender points, dull purplish spots, nodules, or cysts. By using these two therapies, blood can be activated to remove stasis and collaterals can be unblocked to dissolve nodules. Then we should puncture points on the abdomen or low back to strengthen healthy qi.

Normally, the pathogenesis of refractory CLBP is deficient and excess in complexity; moreover, the deficiency of healthy qi is dominant. Based on the TCM theories that a disease should be treated from its root and that a prolonged disease results in deficiency and stagnation, we should initially reinforce the healthy one to eliminate the pathogens. In other words, we should initially puncture tonic points on the abdomen and sometimes add moxibustion simultaneously. When the pain has been partially relieved and the healthy qi is supplemented to a certain extent, we should puncture points on the low back to promote the circulation of qi and blood to further relieve the pain. This treatment strategy conforms to the TCM treatment theory that unblocking obstruction to qi circulation is key to pain relief.

### 4.3. Suitable Combination of Therapies according to Pathogenesis

The filiform needle therapy is widely used in clinical practice to regulate the flow of qi. However, existence of qi is a prerequisite for such treatment. Merely using the filiform needle therapy to treat patients with deficiency of qi, yang, or essence produces the paradoxical effect since no qi can be regulated; instead, healthy qi is consumed. Consequently, the pain still exists after prolonged treatment. Therefore, we should treat CLBP based on the TCM therapeutic principle of observing the pulse and syndromes to treat the disease. For example, if the pain is caused by qi stagnation and blood stasis or if the lumbar function is reduced due to pain, we can use the therapy of acupuncture combined with movement to promote the circulation of qi and activate blood to unblock collaterals. For patients with the deficiency syndrome, we can combine the filiform needle therapy with prescription of Chinese medicine and moxibustion on tonic points on the abdomen. However, selection of tonic points on the abdomen is often disregarded by many practitioners providing clinical treatment.

In line with the TCM theory of chronic diseases transforming to collaterals and causing stagnation, the syndrome of excess resulting from deficiency is common in CLBP. The typical signs are dull purplish spots, red and swollen patches, and musculotendinous nodules, in which the pain exists. Under such circumstances, it is not legitimate to merely use the filiform needle therapy. Therefore, we should combine the filiform needle therapy with other acupuncture techniques. Specifically, we can add the fire needling, round sharp needle, or blade needle therapy to disperse the musculotendinous nodules. In order to dissolve the dull purplish spots and red and swollen patches, we can add the bloodletting therapy to activate blood to dispel stasis and purge fire to relieve swelling. However, since most of these acupuncture therapies are a reducing method, we want to emphasize the importance of using the fire needling therapy because, as a therapeutic principle of simultaneous elimination and reinforcement, it can produce optimum effects on warming and unblocking meridians by dissipating nodules, dispelling blood stasis, expelling cold and dampness, warming yang, and replenishing qi.

Moxibustion can reinforce healthy qi and warm and unblock meridians. Using moxa on a larger area of interest, for example, using moxa along the governor vessel or putting a big moxa box on the painful area, invigorates yang qi and warms and disperses cold-dampness. On the other hand, using direct moxibustion or thermal-sensitive moxibustion on a small and specific area of interest can conduct the flow of meridian qi better. In other words, it can promote transmission sensation along meridians and collaterals to the location of the pain better, and thus, the effect of moxibustion can act better on the affected area. Suspended moxibustion and warming needle moxibustion are preferable to patients with consumption since the way to burn moxa in these two therapies creates mild heat in a gradual way, which accords with the TCM theory of mild fire supplementing qi.

Using acupuncture on extremely weak patients is contraindicated; instead, medication and moxibustion are preferable. Chapter 4 of Lingshu's Miraculous Pivot recommends that people who suffer from yin, yang, or qi deficiency or those who have a weak constitution be prescribed Chinese medicine rather than treated with acupuncture. Accordingly, patients who are elderly, have a weak constitution, suffer from osteoporosis, or whose pain has been relieved to the extent that they have progressed to the lumbar functional recovery phase should be treated with moxibustion and/or prescribed Chinese drugs including Lujiao (Cornu Cervi), jinmaogouji (Cibotium Barometz), shengduzhong (Cortex Eucommiae), xuduan (Radix Dipsaci), sangjisheng (Ramulus Loranthi), tusizi (Semen Cuscutae), gusuibu (Rhizoma Drynariae), jixueteng (Caulis Spatholobi), weilingxian (Radix Clematidis), shengbaizhu (Rhizoma Atractylodis Macrocephalae), and laoguancao (Herba Erodii Seu Geranii). These Chinese drugs can tonify the kidney essence, activate blood to unlock collaterals, and strengthen the spleen to disperse dampness.

## 5. Conclusion

This study has proven that our research approach accords with the TCM theories of individual treatment and syndrome differentiation. In light of the CLBP pathogenesis of deficiency and excess in complexity, especially the complicated pathogenesis of refractory CLBP, the aforementioned acupuncture-moxibustion therapies should be applied appropriately according to each patient's unique pathogenesis. Before applying the reinforcing and circulation-promoting protocol of acupuncture and moxibustion to treat refractory CLBP, we should identify the pathogenesis based on syndrome differentiation. Besides, during treatment, we should give great importance to the therapeutic principle of reinforcing healthy qi and tonifying deficiency. In addition, when the pain has been relieved in a short term, the treatment should not be discontinued. Instead, we should continue to carry out consolidation treatment to reduce the recurrence rate and maintain the efficacy over long periods. However, this study could have been more fruitful if we had invited more patients.

## Figures and Tables

**Table 1:** General information of 24 cases.

Age		Gender		Marital status	
N (missing)	24 (0)	N (missing)	24 (0)	N (missing)	24 (0)
Mean (SD)	53.29 (12.34)	Male *n* (%)	7 (29.17)	Married N (%)	21 (87.50)
Median (Q3-Q1)	53.00 (12.50)	Female N (%)	17 (70.83)	Single N (%)	3 (12.50)
(Q1, Q3)	(48.00, 60.50)				
(Min,max)	(31.00, 78.00)				

**Table 2:** Basic data of diseases in 24 patients.

Name of syndrome in		*N* (%)	Duration of low back pain (month)		Diagnosis in Western medicine	*N* (%)
Low back pain	*N* (Missing)	24 (0)	*N* (Missing)	24 (0)	*N* (Missing)	24 (0)
	No	2 (8.33)	Mean (SD)	71.26 (105.42)	Nonspecific low back pain	0 (0.00)
	Yes	22 (91.67)	Median (*Q*3-*Q*1)	24.00 (91.50)	Soft tissue lesion	1 (4.17)
Pain in lower extremities	*N* (Missing)	24 (0)	*Q*1, *Q*3	(10.50, 102.00)	Lumbar intervertebral disc disease	17 (70.83)
	No	14 (58.33)	(Min, Max)	(0.31, 480.00)	Vertebral column disease	4 (16.67)
	Yes	10 (41.67)			others	2 (8.33)
Pain in gluteal, sacrum or hip	*N* (Missing)	24 (0)				
	No	23 (95.83)				
	Yes	1 (4.17)				
Back pain	*N* (Missing)	24 (0)				
	No	24 (100.00)				
	Yes	0 (0.00)				

**Table 3:** Pain conditions of 24 cases.

Nature of pain		*N* (%)	Induced by		*N* (%)	Relieved by		*N* (%)
Distending pian	*N* (Missing)	24 (0)	Trauma	*N* (Missing)	24 (0)	Rest	*N* (Missing)	24 (0)
	No	10 (41.67)		No	17 (70.83)		No	10 (41.67)
	Yes	14 (58.33)		Yes	7 (29.17)		Yes	14 (58.33)
Stabbing pain	*N* (Missing)	24 (0)	Overuse	*N* (Missing)	24 (0)	Mild exercise	*N* (Missing)	24 (0)
	No	16 (66.67)		No	22 (91.67)		No	16 (66.67)
	Yes	8 (33.33)		Yes	2 (8.33)		Yes	8 (33.33)
Sore pain	*N* (Missing)	24 (0)	Work environment	*N* (Missing)	24 (0)	Warmth	*N* (Missing)	24 (0)
	No	20 (83.33)		No	22 (91.67)		No	8 (33.33)
	Yes	4 (16.67)		Yes	2 (8.33)		Yes	16 (66.67)
Pressing pain	*N* (Missing)	24 (0)	Living environment	*N* (Missing)	24 (0)	Pressing	*N* (Missing)	24 (0)
	No	21 (87.50)		No	13 (54.17)		No	16 (66.67)
	Yes	3 (12.50)		Yes	11 (45.83)		Yes	8 (33.33)
Radiating pain	*N* (Miss-ing)	24(0)	Primary Diseases	*N* (Missing)	24(0)	Not clear	*N* (Missing)	24(0)
	No	15 (62.50)		No	22 (91.67)		No	22 (91.67)
Others	Yes	9 (37.50)	No clear cause	Yes	2 (8.33)		Yes	2 (8.33)
	*N* (Missing)	24 (0)		*N* (Missing)	24 (0)	Others	*N* (Missing)	24 (0)
	No	16 (66.67)		No	16 (66.67)		No	22 (91.67)
	Yes	8 (33.33)	Others	Yes	8 (33.33)		Yes	2 (8.33)
				*N* (Missing)	24(0)			
				No	20 (83.33)			

**Table 4:** Distribution and comparison of treatment and onset time.

Treat-ment period (*d*)		Times of treatment		Onset time		Improvement time	
*N* (Missing)	24 (0)	*N* (Missing)	24(0)	*N* (Miss-ing)	24 (0)	*N* (Missing)	24 (0)
Mean (SD)	42.46 (31.18)	Mean (SD)	9.13 (5.09)	Mean (SD)	9.54 (15.71)	Mean (SD)	14.67 (17.14)
Median (*Q*3-*Q*1)	39.00 (46.00)	Median (*Q*3-*Q*1)	7.50 (8.50)	Median (*Q*3-*Q*1)	3.00 (5.50)	Median (*Q*3-*Q*1)	9.00 (12.50)
*Q*1, *Q*3	(15.50, 61.50)	*Q*1, *Q*3	(5.00, 13.50)	*Q*1, *Q*3	(2.00, 7.50)	*Q*1, *Q*3	(2.50, 15.00)
(Min, Max)	(7.00, 125.00)	(Min, Max)	(3.00, 19.00)	(Min, Max)	(2.00, 70.00)	(Min, Max)	(2.00, 70.00)

**Table 5:** Comparison of observation index before and after treatment.

Groups	Number of cases	VAS	Statistic count (*t*)	*P*	Number of cases	Oswestry disability index	Statistic count (*s*)	*P*
Before treatment	24	6.83 ± 2.18	9.96	*P* < 0.0001	24	28.21 ± 13.06	138	*P* < 0.0001
After treatment	24	2.13 ± 1.45			24	16.63 ± 7.20		

## Data Availability

All data are included within the article.
